# Genetic duplication of tissue factor reveals subfunctionalization in venous and arterial hemostasis

**DOI:** 10.1371/journal.pgen.1010534

**Published:** 2022-11-30

**Authors:** Steven J. Grzegorski, Yakun Zhao, Catherine E. Richter, Chia-Jui Ku, Kari I. Lavik, Divyani Paul, James H. Morrissey, Jordan A. Shavit

**Affiliations:** 1 Department of Pediatrics, University of Michigan, Ann Arbor, Michigan, United States of America; 2 Department of Biological Chemistry, University of Michigan, Ann Arbor, Michigan, United States of America; 3 Department of Human Genetics, University of Michigan, Ann Arbor, Michigan, United States of America; University of Chicago Medical Center: The University of Chicago Medicine, UNITED STATES

## Abstract

Tissue factor (TF) is an evolutionarily conserved protein necessary for initiation of hemostasis. Zebrafish have two copies of the tissue factor gene (*f3a* and *f3b*) as the result of an ancestral teleost fish duplication event (so called ohnologs). *In vivo* physiologic studies of TF function have been difficult given early lethality of TF knockout in the mouse. We used genome editing to produce knockouts of both *f3a* and *f3b* in zebrafish. Since ohnologs arose through sub- or neofunctionalization, they can unmask unknown functions of non-teleost genes and could reveal whether mammalian TF has developmental functions distinct from coagulation. Here we show that a single copy of either *f3a* or *f3b* is necessary and sufficient for normal lifespan. Complete loss of TF results in lethal hemorrhage by 2–4 months despite normal embryonic and vascular development. Larval vascular endothelial injury reveals predominant roles for TFa in venous circulation and TFb in arterial circulation. Finally, we demonstrate that loss of TF predisposes to a stress-induced cardiac tamponade independent of its role in fibrin formation. Overall, our data suggest partial subfunctionalization of TFa and TFb. This multigenic zebrafish model has the potential to facilitate study of the role of TF in different vascular beds.

## Author summary

Tissue factor (TF) is a critical component in the initiation of blood coagulation. It is exposed upon blood vessel injury, which leads to blood clot formation and cessation of hemorrhage. TF is functionally conserved and likely required for survival across all vertebrate species. Due to an ancestral genomic duplication event in the teleost class of fish, zebrafish have two copies of tissue factor (referred to as ohnologs) on separate chromosomes. We exploited this aspect of the zebrafish model to study real time developmental phenotypes previously suggested in mouse studies, with the surprising finding that the *f3* ohnologs have evolved separate functions. We saw no evidence for TF in blood vessel formation, however, we did see an obvious role in protecting the integrity of the vascular space around the heart. We found that clotting was completely blocked in the total absence of TF, but loss of each individual ohnolog led to differential deficiencies in the arterial and venous circulations. This type of evolution is known as “subfunctionalization” and presents an opportunity to further understand differences between blood clot formation based on vascular location.

## Introduction

Preventing hemorrhage in the setting of normal physiology requires a well-regulated plasma coagulation system. Under normal conditions, the potent activator tissue factor (TF, gene symbol *F3*) is expressed as a transmembrane glycoprotein on the surface of many cell types outside the vasculature. Disruption of the endothelium exposes TF which binds to the circulating zymogen, factor VII (FVII, 99% of circulating factor), or its active protease (FVIIa, 1%) [1]. Once bound, FVII is rapidly activated to FVIIa and the TF/FVIIa complex serves as an initiator of coagulation via the activation of factor X and factor IX [2, 3]. Apart from vascular damage, infection induces proinflammatory cytokines known to trigger the expression of TF on the surface of mononuclear and endothelial cells [4]. Additionally, many neoplasms express high levels of TF which can enter circulation through the shedding of microvesicles [5].

Beyond hemostasis, the TF/FVIIa complex has a role in cell signaling through cleavage of G-protein coupled, protease activated receptors (PARs) [6] and interaction with β_1_ integrins [7, 8]. Several tissue-specific knockouts of *F3* have been described in mice, including Mlc2v-cre [9], LysM-cre, Tie2-cre [10], Nestin-cre [11], and K14-cre [4]. Complete global TF deficiency leads to lethality by embryonic day 8.5–10.5, making it the most severe of procoagulant knockouts [12–14]. In contrast, loss of FVII results in neonatal lethality likely due to diffusion of FVII from the maternal to embryonic circulation [15, 16].

Due to the early lethality of complete *F3* knockout, a human *F3* minigene was introduced into *F3*^-/-^ mice, resulting in TF protein levels 1–2% of normal [17]. Although the minigene rescues survival into adulthood, low TF mice exhibit prolonged bleeding after hemostatic challenge and have increased rates of hemorrhage. Interestingly, while human TF activates coagulation in mice, mouse TF has about a six-fold lower activity in activation of coagulation in human plasma [18]. This is due to species-specific interaction between TF and FVIIa [19, 20]. Therefore, an accessible model that does not rely on the complications of xenogeneic proteins is critical for understanding TF deficiency.

The zebrafish is a small aquatic vertebrate with a highly conserved hemostatic system [21, 22], high fecundity, and rapid, transparent, and external development. Circulating blood is visible by 36 hours of life and assays for assessing coagulation *in vivo* are readily performed between 72 and 120 hours post fertilization (hpf) [23, 24]. Importantly, zebrafish are able to survive severe hemostatic defects that are usually lethal in mammals, and develop into adulthood [25–29]. Zebrafish have two copies of *f3* (*f3a* and *f3b*) due to an ancestral whole genome duplication event (referred to as “ohnologs”) [30]. Previously performed antisense knockdowns of *f3a* and *f3b* in zebrafish suggest a role in early vascular development and hemostasis [31, 32], although the morpholino-based knockdown technology is known for off-target effects, especially in the vasculature [33]. To our knowledge, no studies have reported the consequences of a total loss of TF activity in zebrafish, nor have studies previously genetically knocked out the ohnologs individually and in combination.

Although the first genetic heterozygous deficiency of *F3* in humans has recently been described, [34] complete loss has never been seen in humans and is difficult to study in mice. Here we show the use of genome editing with CRISPR/Cas9 to generate loss-of-function alleles in both copies of zebrafish *f3*. Complete loss of TF is compatible with embryonic through juvenile development but leads to early adult lethality, although a single allele of either ohnolog is sufficient to enable survival into adulthood. Furthermore, we show that TFa has higher procoagulant activity than TFb *in vitro* and is sufficient for venous hemostasis, while TFb is sufficient for arterial coagulation. Understanding the spatial and temporal control of *f3a* and *f3b*, as well as their functional differences, could prove valuable for understanding regulation of the coagulation cascade, as well as identifying potential distinct or novel activities of TF in mammalian hemostasis.

## Results

### Characterization of the *f3a* and *f3b* genes

Analysis of the Refseq database [35] revealed two orthologs of *F3*, annotated as *f3a* and *f3b*, with each consisting of 6 exons. *f3a* (NM_001245967.1) is a 13 kilobase (kb) locus on chromosome 24 and *f3b* (NM_001017728.2) is a 7 kb locus on chromosome 2. The coding regions of each gene were isolated from embryonic mRNA and confirmed by Sanger sequencing. The predicted amino acid sequences of TFa (294 aa) and TFb (288 aa) were used for homology analysis (S2 Table). The mature (leader peptide removed) TFa and TFb proteins share 45.2% sequence identity with each other and 34.5% and 32.9% sequence identity with human TF (NP_001984.1), respectively. Protein sequence alignment demonstrates several highly conserved regions (S1 Fig).

Humans have a well-known alternative splice form of *F3*, [36] but no evidence for alternative splicing was found for either TF ortholog in available sequence databases. Human TF is glycosylated at asparagines 11, 124 and 137. [2] Both TFa and TFb contain a homologous asparagine to human Asn11. However, only TFb has a predicted Asn near 137 and neither has a predicted glycosylation site near 124. [37] Additionally, TFa and TFb have 2 predicted conserved disulfide bonds in the extracellular domain and a conserved cysteine in the cytoplasmic tail that is palmitoylated in humans (S1 Fig). [38] Only TFb has potential sites of phosphorylation in the cytoplasmic tail according to PhosNet prediction. [39]

### Genome editing to generate *f3a* and *f3b* null alleles

**Fig 1:**
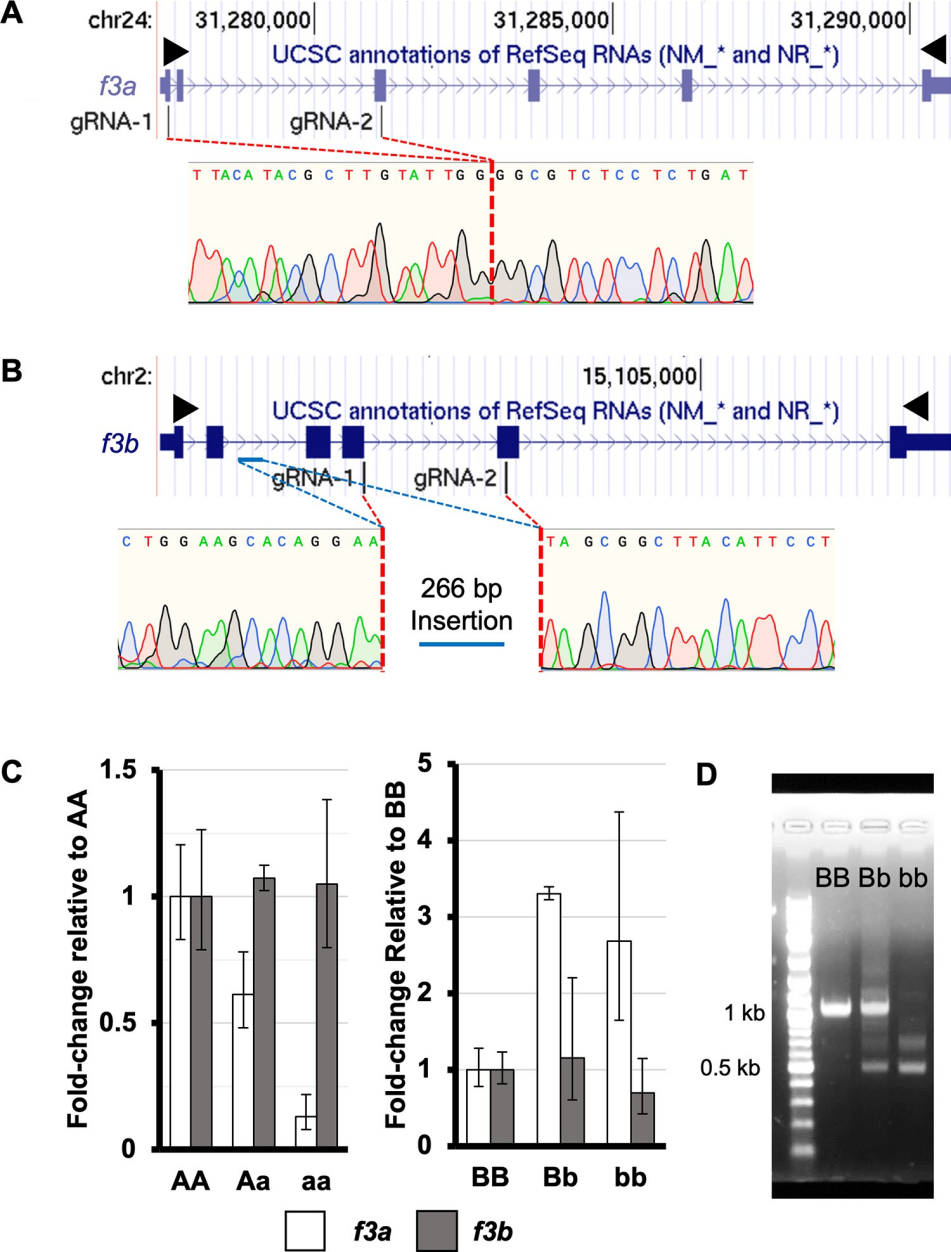
Genome engineering creates large loss-of-function deletions in both copies of zebrafish *f3*. Two sgRNAs each for *f3a* and *f3b* were complexed with Cas9 and injected into single cell embryos. (A) A large deletion was identified between exon 1 and exon 3 in *f3a* resulting in a nonsense mutation. (B) A deletion was introduced between exon 4 and exon 5 of *f3b*. During endogenous repair, a section of intronic DNA (blue bar) was inverted and inserted at the site of the deletion introducing a premature stop codon. Black arrowheads indicate the locations of primers used for full length cDNA amplification. (C) RT-qPCR at 4 dpf demonstrated that the mutant *f3a* allele resulted in lower mRNA levels (consistent with nonsense mediated decay of the residual transcript) without affecting *f3b* levels. Conversely, loss of *f3b* induces upregulation of *f3a*, but the levels of *f3b* transcript were not diminished. (D) *f3b* cDNA was isolated from pools of 4 dpf larvae from heterozygous mutant incrosses. The expected wild-type band was visible in the *BB* and *Bb* mutants. Two smaller bands were evident in the *Bb* and *bb* pools, consistent with alternative splicing of the mutant transcript.

Large stably transmitting deletions were genetically engineered in *f3a* and *f3b* using CRISPR/Cas9 (Fig 1A and 1B). For simplicity, when discussing both *f3* genotypes, wild-type alleles will be referred to as “A” and “B” while null-alleles will be referred to as “a” and “b,” e.g., double heterozygotes are *Aa/Bb* (A* will represent *AA* or *Aa*, B* will represent *BB* or *Bb*). qRT-PCR at 4 dpf (days post fertilization) demonstrated that homozygosity of the *f3a* deletion resulted in reduction of mRNA levels consistent with nonsense-mediated decay (Fig 1C). The *f3b* allele developed a large insertion at the site of editing. Conversely, qRT-PCR of homozygous *f3b* larvae revealed levels of residual transcript consistent with wild-type and heterozygous genotypes and compensatory induction of *f3a* (Fig 1C). When the start codon to the 3’ UTR of the *f3b* transcript was amplified using a single primer pair and analyzed via agarose gel electrophoresis, multiple small transcripts were identified in heterozygous and homozygous *f3b* mutants (Fig 1D). When this mixed pool of products was analyzed using Sanger sequencing, the most prevalent was a splice variant from exon 2 to exon 6 encoding a 93 amino acid peptide which includes the first 65 N-terminal residues of the preprotein and 28 out of frame residues from the last exon. The engineered alleles for both *f3a* and *f3b* result in significant deletions and frameshift mutations that likely generate complete loss of functional TF protein.

### A single *f3* allele is both necessary and sufficient for long term survival

**Fig 2:**
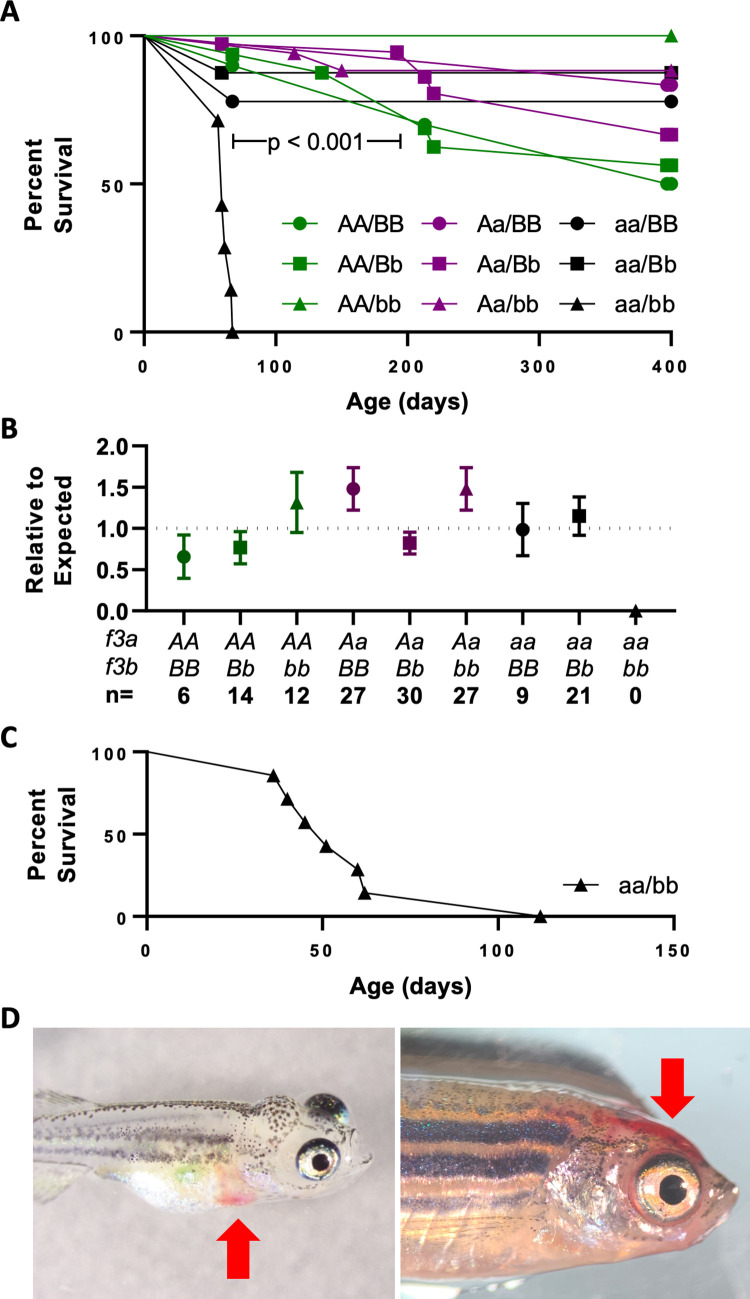
Complete loss of TF activity results in early lethality due to hemorrhage. (A) Fish from a double heterozygous (*Aa/Bb*) *f3* incross were genotyped at 8 weeks of age and found to have normal Mendelian ratios (n = 135, AA/BB = 10, AA/Bb = 17, AA/bb = 7, Aa/BB = 15, Aa/Bb = 36, Aa/bb = 20, aa/BB = 10, aa/Bb = 13, aa/bb = 9). Survival curves show that by 9 weeks, there was statistically significant loss of *aa/bb* offspring. By 400 days, there was a relatively even reduction of the remaining genotypes which is common in wild-type fish, and confirms that a single copy of *f3a* or *f3b* is sufficient for survival. (B) A repeat cohort was left undisturbed until genotyping at 6 months of age. The statistically significant loss of *aa/bb* confirms the early lethality of TF loss independent of genotyping stress. (C) A group of 7 double homozygous mutants were identified at 1 month of age and observed over the following 3 months. 6/7 were lost by 2 months with the single survivor passing at 112 days. (D) Early lethality was due to gross hemorrhage. Examples include the pericardial space (left) and head (right) (red arrows).

A large group of offspring produced from incrossing *Aa/Bb* parents was genotyped at 8 weeks revealing no deviation from the expected Mendelian ratios. However, all 9 double homozygous mutant fish (*aa/bb*) died over the next week (Fig 2A). The remaining genotypes, including *Aa/bb* and *aa/Bb*, demonstrated equivalent survival over the following year (p > 0.05). A second group was raised to 6 months of age before genotyping to eliminate the impact of genotyping stress on survival (Fig 2B). No double homozygous mutant fish were identified in this cohort, consistent with complete TF deficiency being incompatible with long-term survival. A final cohort of 7 *aa/bb* fish was identified at <1 month of age and followed. Six succumbed to hemorrhage by 2 months of age while the last reached sexual maturity before dying at 120 days (Fig 2C). The cause of death across all groups appeared to be overt hemorrhage in the head or pericardial space (Fig 2D). When groups of offspring were generated from *Aa/bb* males crossed to *aa/Bb* females and *Aa/bb* females crossed to *aa/Bb* males, all possible genotypes were recovered at 1 month of age, indicating that maternal deposition of *f3a/f3b* transcripts does not appear to influence early survival.

### Loss of TF does not alter vascular development

**Fig 3:**
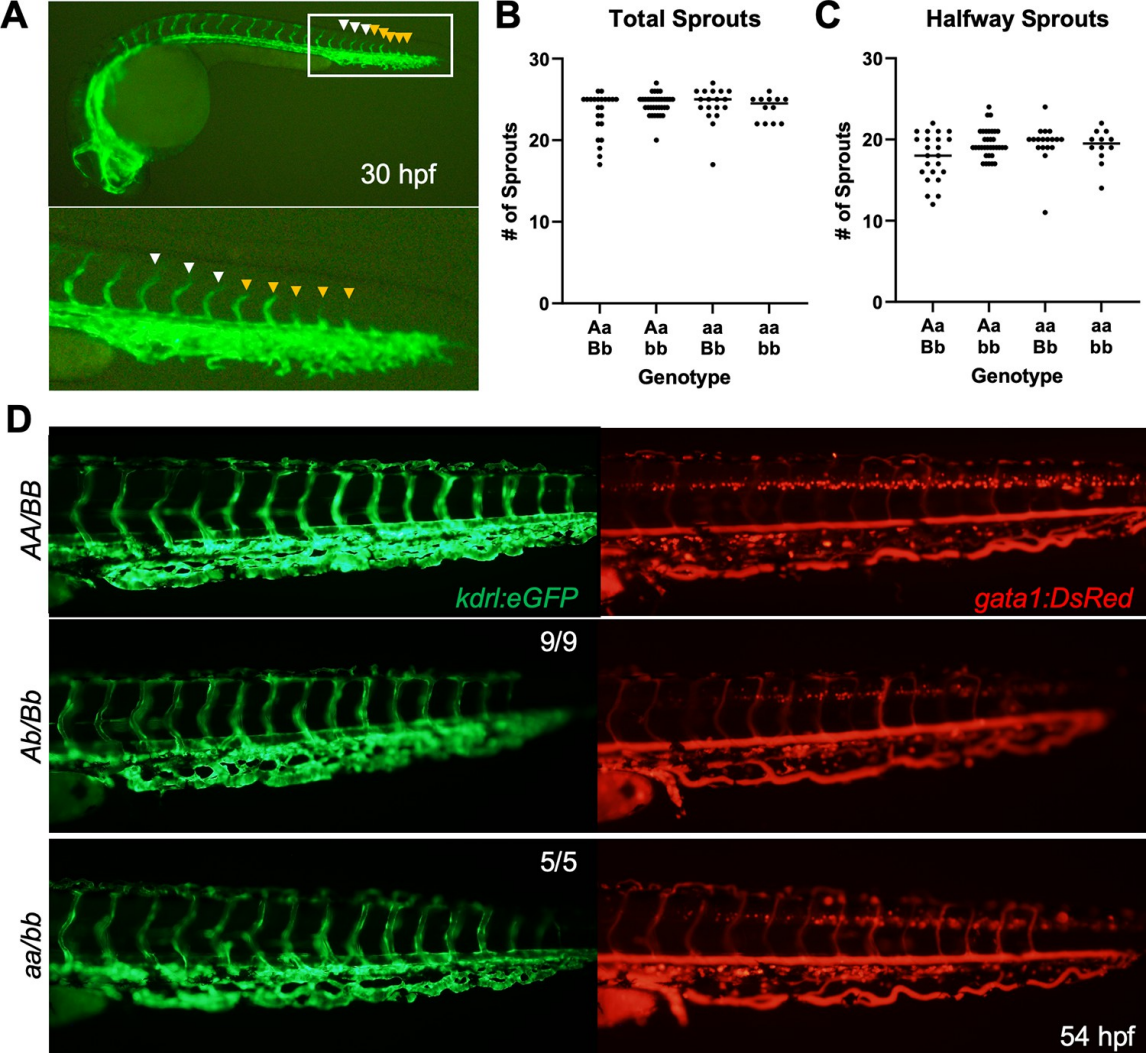
Vascular development is unaffected in the absence of TF. (A) Example of a 30 hpf larva carrying the *kdrl*:*eGFP* transgene marking vascular endothelial cells (top) with a close-up (below). White arrowheads mark examples of vessel sprouts that have made it at least halfway through the expected migration. Orange arrowheads represent sprouts that have not yet passed the halfway point. No statistically significant changes were observed between groups in total vessel sprouts (B) or sprouts that have made it past the halfway point (C) by Mann-Whitney U testing. (D) At 54 hpf, no observable changes are seen in endothelial cell development (left, green) or in vascular integrity (right, red). The latter is evidenced by long exposure in the *gata1*:*DsRed* transgenic background in which erythrocytes are marked by red fluorescence. Representative images for 14 experimental mutant larvae that were scored by a blinded observer are shown along with a wild type control.

Hypothesizing a role of TF in angiogenesis, the *gata1a*:*DsRed* [40] (labeling erythrocytes), and *kdrl*:*eGFP* [41] (labeling endothelial cells) transgenic lines were used to investigate endothelial development and vascular perfusion. At 30 hpf, the number of vascular sprouts and their growth progress were scored prior to genotyping, and no significant difference was observed across genotypes (Fig 3A–3C). At 54 hpf, there were no obvious defects in endothelial development or erythrocyte perfusion in *aa/bb* larvae (Fig 3D). No obvious morphological defects were seen in the first week of life under bright field examination. Overall, these data indicate that TF is not necessary for the differentiation, proliferation, or function of the vascular endothelium in zebrafish.

### *f3a* and *b* demonstrate tissue-specific roles in venous and arterial hemostasis

**Fig 4:**
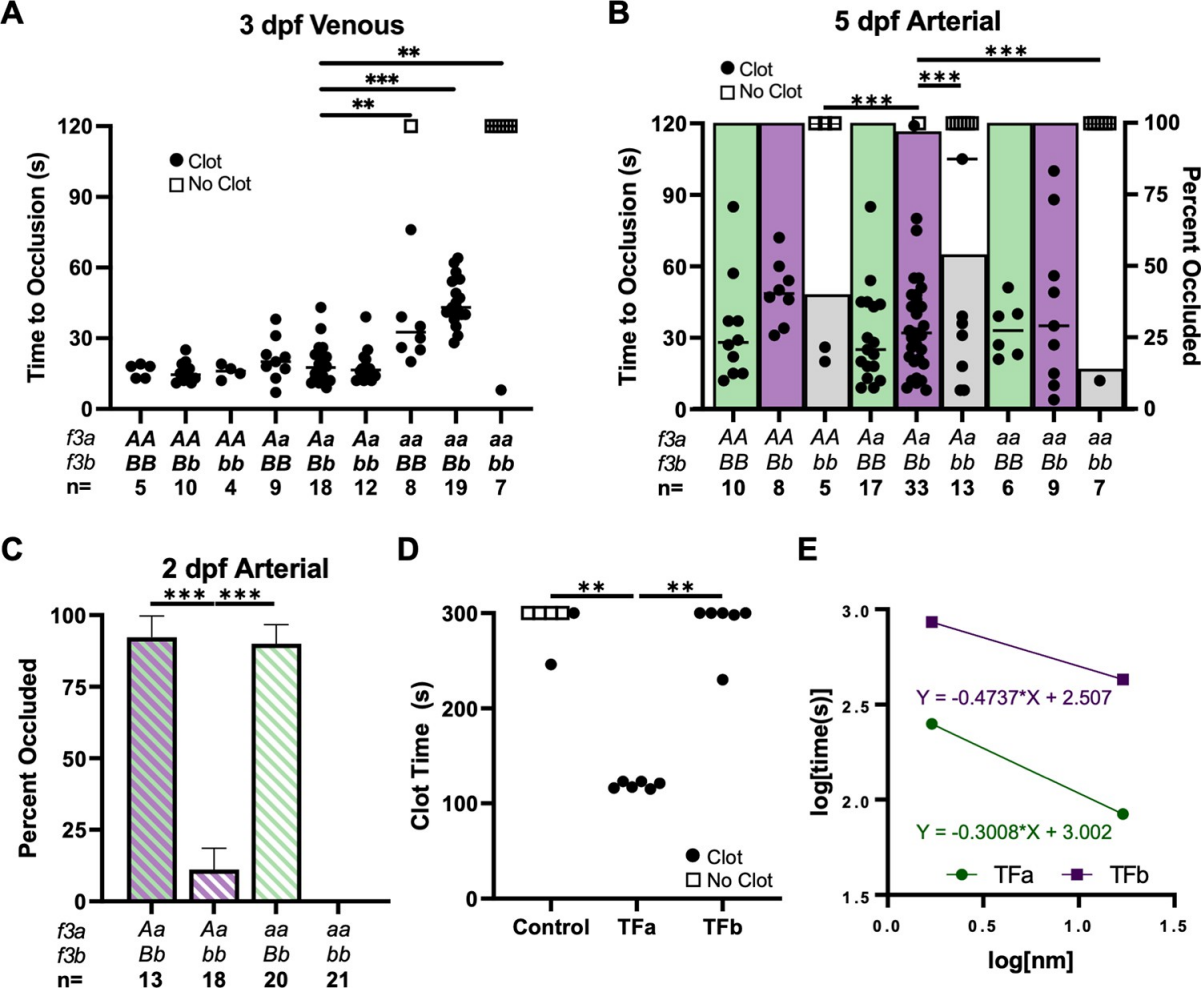
TFa and TFb have evolved partial subfunctionalization according to *in vivo* vascular endothelial laser injury model and *in vitro* recombinant protein clotting assays. (A) Laser-mediated venous endothelial injury at 3 dpf shows that the time to occlusion (TTO) was not altered by loss of TFb (*AA/bb*, *Aa/bb*) but was delayed in the complete absence of TFa (*aa/BB*, *aa/Bb*). Thrombus formation was absent after complete TF loss (*aa/bb*). (B) Laser-mediated arterial endothelial injury at 5 dpf shows that the ability to form occlusive thrombi was significantly reduced in fish lacking TFb (*AA/bb*, *Aa/bb*, *aa/bb*, indicated by grey bars) compared to *BB* and *Bb* groups (green and purple bars; respectively). (C) Endothelial injury at 2 dpf confirms the dependence on TFb in the arterial system prior to the presence of thrombocytes. Occlusive thrombi were nearly absent without TFb (*Aa/bb*, *aa/bb*). (D) Relipidated recombinant TFa or TFb proteins (173 nM) were mixed with citrated trout plasma, and recalcified. TFa-liposomes induced clot formation during a manual tilt test significantly faster than TFb-liposomes or empty liposome controls. The reaction was observed until 300 seconds, and then again at 15 minutes to assess for delayed clot formation (the latter observation is indicated by an open or closed circle at 300 seconds). (E) The assay was repeated with relipidated TF diluted in 0.5x HBS (20 mM HEPES pH 7.5, 50 mM NaCl) and plotted on log-log axes demonstrating 10 to 100-fold higher procoagulant activity of TFa over TFb. Statistical significance by Mann-Whitney *U* testing: ** < 0.01, *** < 0.001. All phenotypic data were collected by an observer blinded to genotype. Open squares in A and B indicate individual larvae that did not occlude within 120 seconds.

Laser-induced vascular endothelial injury is a methodology that injures the endothelium leading to exposure of TF, and thereby examines the ability of the coagulation cascade to generate a proper hemostatic response. This was used to trigger thrombus formation in the venous system at 3 dpf in *f3* mutants (Fig 4A). Loss of TFb alone did not lead to any alterations in the time to occlusion (TTO). Loss of TFa resulted in a mild delay in median TTO from <20 to 32 seconds. In the absence of TFa, heterozygosity for *f3b (aa/Bb)* exacerbated the TTO delay to 43 seconds while complete deficiency of TF (*aa/bb)* resulted in the almost complete inability to form occlusive thrombi (Fig 4A, open squares). Thus, a single copy of either *f3a* or *f3b* is necessary for venous occlusion, but only *f3a* is sufficient for normal TTO.

Laser-mediated endothelial injury was performed on the dorsal aorta at 5 dpf to determine the role of the *f3* ohnologs in arterial hemostasis. Loss of *f3b* but not *f3a* led to an increased median TTO. Notably, complete loss of *f3b* led to a statistically significant increase in failure to occlude with phenotypic penetrance of 60%, 46% and 86% in *AA/bb*, *Aa/bb*, and *aa/bb* offspring; respectively (Fig 4B). When subset for *f3b-null* larvae that occluded, the median TTO was consistent across genotypes, suggesting a binary phenotype. Endothelial injury was then performed at 2 dpf, prior to the robust expansion of thrombocytes in circulation [42, 43]. Greater than 90% of *Aa/Bb* and *aa/Bb* larvae formed occlusive clots following arterial damage. In contrast, significantly fewer *Aa/bb* (11%) and *aa/bb* (0%) larvae formed occlusive thrombi (Fig 4C). Therefore, in contrast to the stochastic role of *f3a* in venous thrombus formation, one allele of *f3b* appears to be necessary and sufficient for arterial thrombus formation.

### TFa demonstrates greater in vitro plasma procoagulant activity than TFb

The differential responses in the venous and arterial vasculature suggest a possible functional difference between the two ohnologs. One method for testing this hypothesis is through examination of fibrin formation in platelet-poor plasma *ex vivo*, a common technique used in mammalian studies of hemostasis. This could determine if there are functional differences between TFa and TFb proteins. We have found that as in mammals, zebrafish venous hemostasis is more dependent on fibrin, rather than thrombocytes (platelets in mammals). We could not consistently isolate enough plasma from zebrafish, so we obtained thrombocyte-poor trout plasma and performed a manual tilt-test after activation by liposomes containing recombinant TFa or TFb proteins (Fig 4D). Plasma activated by TFa-liposomes clotted in roughly 120 seconds. Samples initiated with TFb-containing liposomes were not observed to form clots until a final 15-minute examination point. A titration of liposomes at a lower sodium concentration demonstrated roughly 10 to 100-fold higher procoagulant activity of TFa-liposomes relative to TFb-liposomes (Fig 4E), demonstrating a functional difference between the two proteins.

### Loss of factor IX (FIX) modifies clot formation in *f3a*-null offspring

Zebrafish lack factor XI (FXI), raising the possibility that FIX (gene symbol *f9b*) activation is completely dependent on TF activity and that the previous phenotypic variabilities could represent differential substrate preference for TF:FVIIa complexes containing TFa versus TFb. Offspring from *Aa/Bb*; *f9b*^+/-^ incrosses were subjected to endothelial injury at various time points and sub-analysis of relevant genotypes explored (full dataset available in S2 Fig).

**Fig 5:**
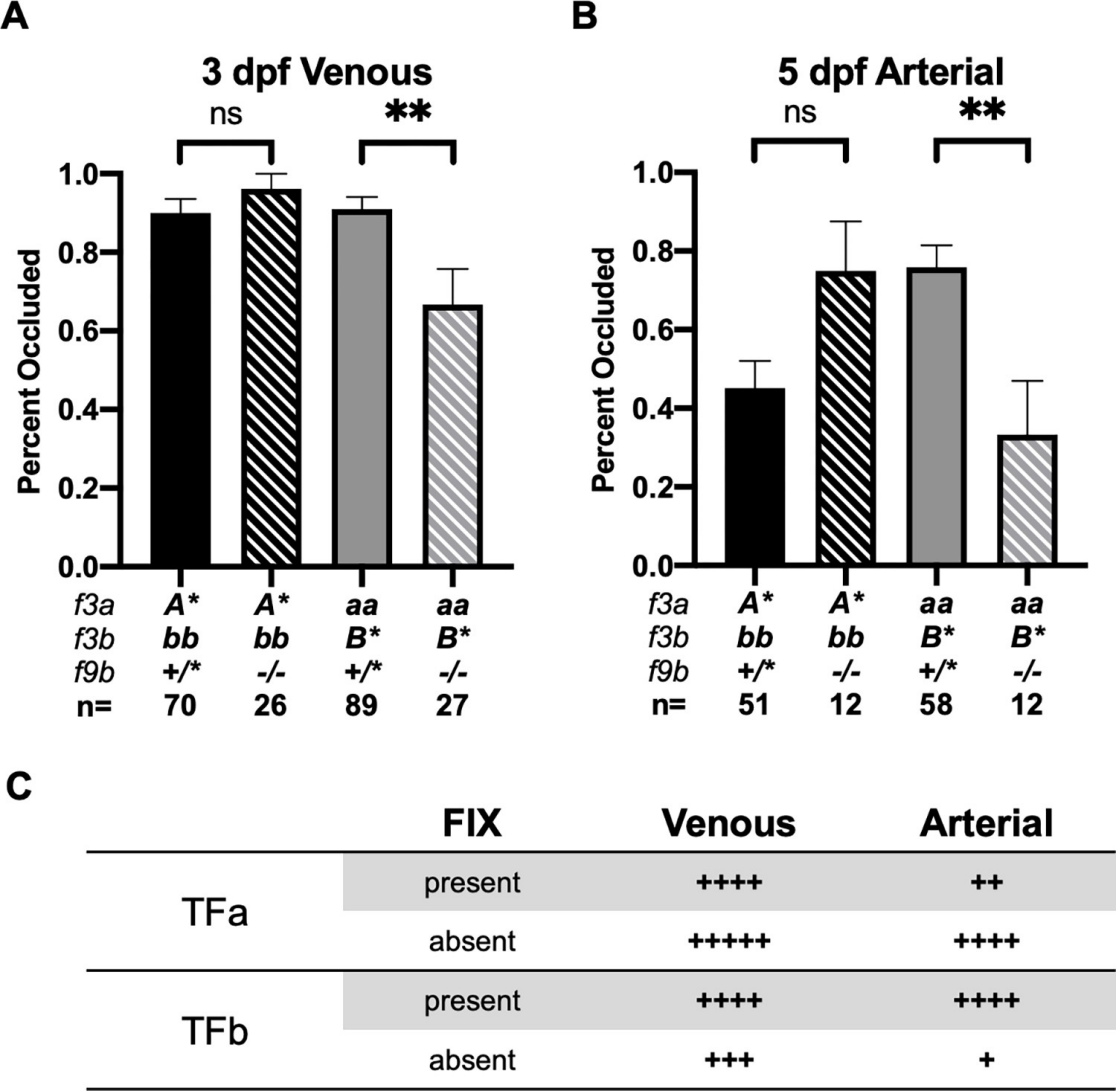
Loss of FIXb impairs clot formation in *f3a*-null offspring. (A) Loss of *f9b* does not influence induced venous occlusion in A*/bb but does decrease total frequency of occlusion in aa/B* larvae at 3 dpf. (B) Loss of *f9b* does not significantly affect clot formation in A*/bb offspring. In aa/B* larvae, loss of *f9b* leads to a significant impairment of occlusive clot formation. Statistical significance by Mann-Whitney *U* testing: ** < 0.01. A* represents *AA* or *Aa*, B* represents *BB* or *Bb*. (C) Summary of data in (A) and (B) with proposed relative ohnolog occlusive activity in the presence and absence of FIX, in arterial versus venous systems.

At 3 dpf, *f9b* genotype did not influence the ability to form occlusive venous clots in *f3b-*null offspring. In contrast, complete loss of *f9b* in *f3a-*null larvae resulted in a significant reduction in clot frequency to 67% compared to 91% in *f9b*^+/*^ offspring (p<0.01; Fig 5A). These data suggest that venous clot formation is primarily mediated from canonical activation of FX by TF/FVII. The requirement for FIX in the absence of TFa, and not the absence of TFb, supports the hypothesis that TFb has lower efficiency in the venous system, thus necessitating FIX-mediated amplification.

At 5 dpf, *f3b-*null offspring formed occlusive arterial clots only 45% of the time when carrying at least 1 copy of *f9b*. This went up to 75% when both copies of *f9b* were lost, although not significant (p = 0.06). In contrast, *f3a*-null offspring saw a significant decrease in clot formation from 76% of *f9b*^+/*^ larvae to 33% of *f9b*^*-/-*^ offspring (p < 0.01; Fig 5B). Overall, FIX significantly enhances TFb-mediated clot formation with a larger effect in the arterial circulation (Fig 5C).

### Loss of TFb partially protects against inherited thrombophilia

Our previous studies demonstrate that the loss of antithrombin (AT3) in zebrafish results in spontaneous venous thrombosis at 3 dpf with subsequent disruption of induced clot formation due to hypofibrinogenemia. This is followed by early adult lethality due to intracardiac thrombosis [29].

**Fig 6:**
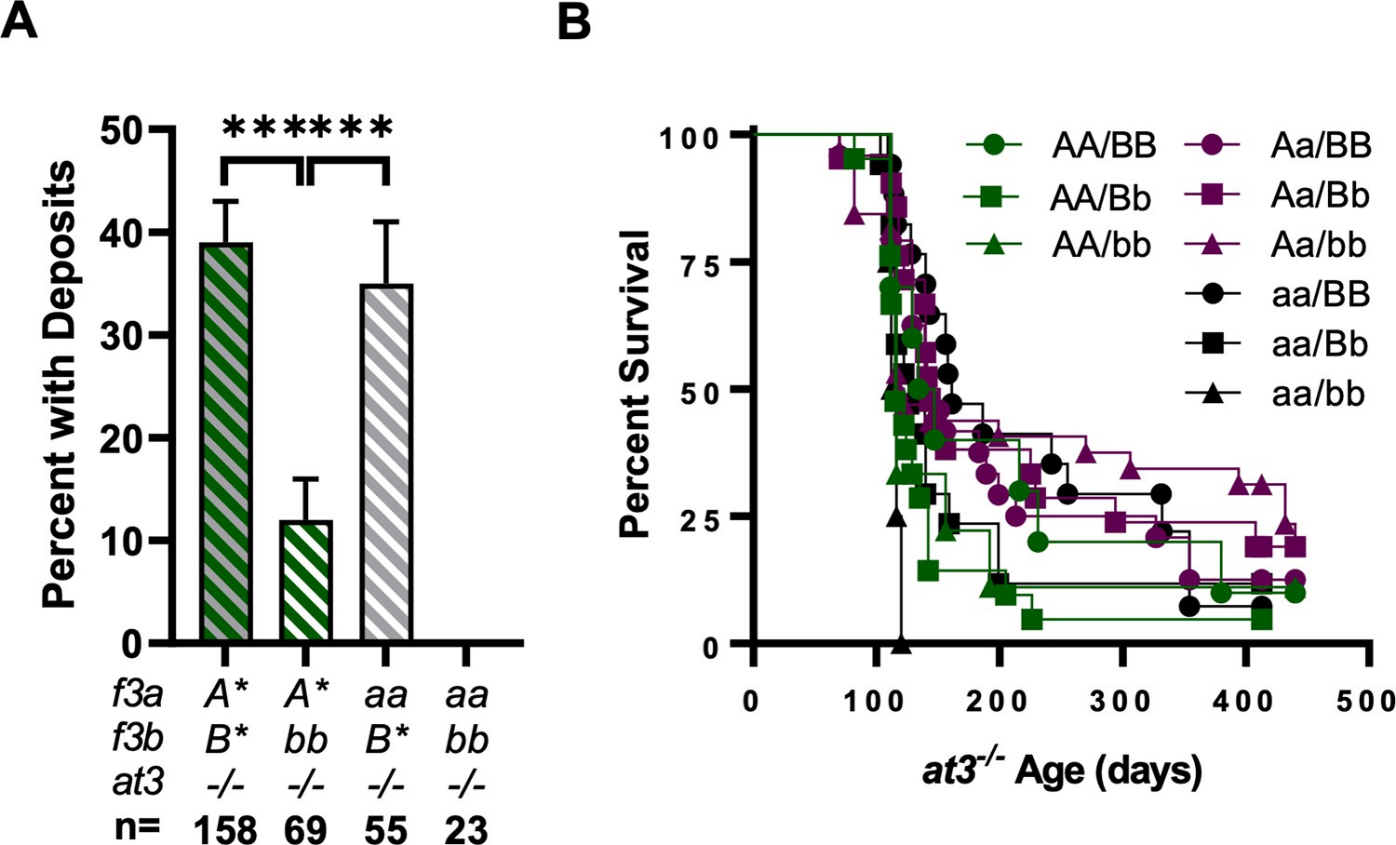
Loss of TFb protects against spontaneous thrombosis in *at3*^*-/-*^ larvae but does not improve long term survival. (A) Fibrin deposits were assessed at 5 dpf. Deficiency of TFa did not alter thrombosis scores while loss of TFb led to less deposition (B) When followed for >1 year, *f3* genotype did not influence *at3*^*-/-*^ survival; however, all *aa/bb* offspring were lost by 120 days, consistent with TF dependent lethality. Statistical significance by binomial proportion test: *** < 0.001. A* represents *AA* or *Aa*, B* represents *BB* or *Bb*.

A cohort of *at3*^*-/-*^*;Aa/Bb* fish carrying a GFP-tagged fibrinogen beta transgene were incrossed and their offspring scored for spontaneous venous thrombosis at 5 dpf, followed by *f3* genotyping. 39% of larvae containing at least one copy of each *f3* (A*/B*) developed thrombosis. This was not statistically different from the 36% frequency of *f3a*^*-/-*^ larvae with at least one copy of *f3b* (aa/B*). However, *f3b*^*-/-*^ larvae with at least one copy of *f3a* (A*/bb) had a significantly lower rate of thrombosis at 12%. *aa/bb* larvae did not have evidence of thrombosis (Fig 6A). These data indicate spontaneous venous thrombosis in the context of AT3 deficiency is predominantly the result of *f3b* activity.

Additional offspring from the *at3*^*-/-*^*;Aa/Bb* incross were followed for survival and genotyped at 2–3 months of age. There were no clear differences in survival by genotype through the first 400 days aside from the early expected loss of *aa/bb* (Fig 6B). This shows that TFb-mediated modification of early acute spontaneous thrombosis in *at3*^*-/-*^ larvae does not translate into protection against long-term adult lethality.

### Loss of TF predisposes to cardiac tamponade independent of fibrin formation

**Fig 7:**
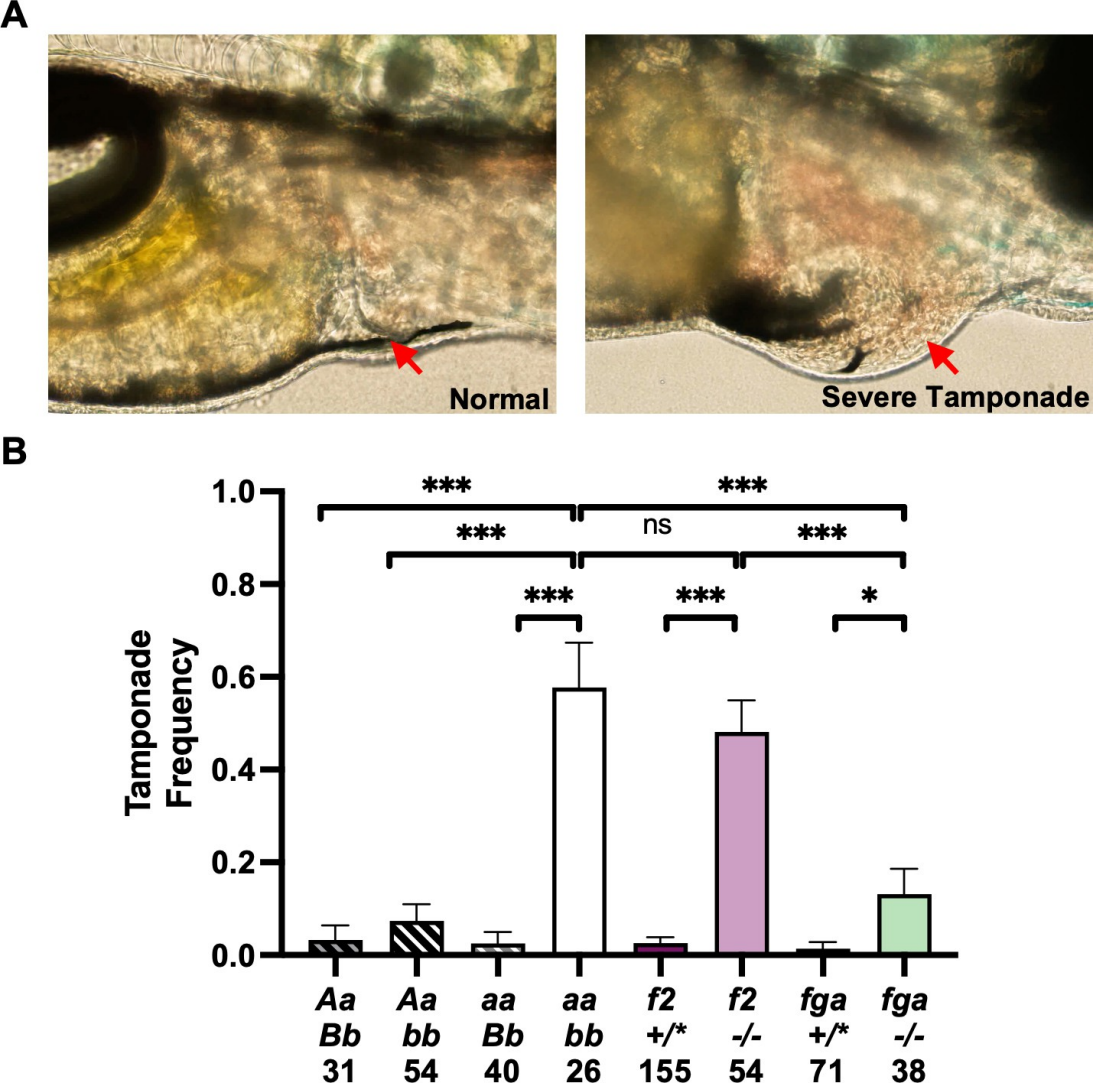
Loss of TF contributes to risk of cardiac tamponade. (A) Stress was chemically induced in 3 dpf larvae by exposure for 16 hours using 25 μM epinephrine and 0.01% hydrocortisone. Larvae were scored for the presence of erythrocytes in the pericardial space (noted by red arrows) by an observer blinded to genotype. Examples of control (left) and tamponade-positive fish (right) are shown. (B) TF-, prothrombin (*f2*)- and fibrinogen (*fga*)-deficient fish all showed significantly elevated rates of tamponade relative to controls. Furthermore, TF- and prothrombin-deficient larvae had comparable rates of tamponade and are both significantly increased relative to *fga* mutants. Statistical significance by binomial proportion test: * < 0.5, ** < 0.01, *** < 0.001. +/* represents +/+ or +/-.

The laboratory aquatic milieu, relatively low blood pressures, and lack of birthing trauma create a low stress early developmental environment for zebrafish compared to mammals. To understand if these factors explain the ability of zebrafish to survive to 2 months of age without TF, a chemical stress challenge was initiated at 3 dpf. After 16 hours of treatment with cortisol and epinephrine, the hearts of treated fish were scored by a blinded observer (Fig 7A and S1 and S2 Video). Zebrafish with overall TF deficiency developed cardiomegaly and a concomitant significant increase in erythrocytes in the pericardial space, consistent with cardiac tamponade (Fig 7B, left). Prothrombin-deficient fish exhibited a similar frequency of cardiac tamponade (Fig 7B, center). However, fibrinogen-deficient larvae were unchanged from wild-type (Fig 7B, right). These data suggest that the risk of tamponade is related to the roles of TF and prothrombin in thrombocyte activation and/or non-hemostatic signaling.

## Discussion

Study of the evolution of the coagulation cascade has been enhanced with increased access to novel model organisms and their genomes [44–46]. The function of TF is essential for vertebrate life despite its low sequence level conservation (~60% between human and mouse and ~40% between human and fish), and it is conserved even in jawless fish. TF in lamprey shares the same broad structure with humans, mice, and zebrafish, including an extracellular domain comprising two fibronectin type III modules, a transmembrane domain, and a cytoplasmic tail. Simulations suggest that despite the low sequence homology between humans and lamprey, the dynamics of TF activation of FVII are conserved [47]. It’s safe to reason that these same dynamics likely apply to the two copies of TF in zebrafish, especially given demonstrated sequence level conservation of disulfide bridges and glycosylation sites. The low degree of sequence conservation has been a challenge for the study of TF, necessitating development of intricate *in vitro* systems and specialized reagents. While loss of TF in mice has been linked to abnormalities in development, angiogenesis, inflammation, and regeneration, it has been difficult to validate these phenotypes and eliminate artifacts in whole-organism models [4, 13, 48, 49]. Zebrafish offer a model to address many of these concerns due to their easily visualized development, conserved coagulation cascade, resistance to hemostatic imbalance, and uniquely due to the presence of 2 distinct genomic copies of *f3*. Importantly, while this study focuses on phenotypes attributed to the extracellular domain of TF, there are changes in the intracellular domain that are not addressed. For example, phosphorylation of the cytoplasmic tail may contribute to TF influenced intracellular signaling. Only TFa demonstrates conserved sequence suggesting that this phosphorylation could occur.

Prior studies of TF in zebrafish have focused on the use of morpholino antisense technology to transiently knock down translation in larvae. Knockdown of either *f3a* or *f3b* using morpholinos appears to cause angiogenic defects, including vascular leak of small molecules and delay of venous angiogenesis not observed in this study. [31, 32, 50] In contrast to those studies, we have employed genetic mutants to study simultaneous, complete, and permanent deficiency of TFa and TFb. We found that zebrafish lacking TF typically survive to 8 weeks of age without gross defects in vascular morphology or endothelial development. We believe our large engineered genomic deletions reduce the likelihood of nucleic acid based genetic compensation [51] as an explanation and increases the likelihood that the observed compensation is driven by protein level homeostasis. The gross morphologic phenotypic differences observed in the studies using morpholinos to knock down TF may be due to a combination of developmental delays and toxicity due to off target effects inherent in morpholino experiments. However, subtle vascular-specific phenotypes could have been missed in our analyses.

We found that complete TF deficiency typically causes lethality by 2 months (early adulthood), but a single allele of either *f3a* or *f3b* is sufficient for normal survival and sexual reproduction. These data confirm that although TFa and TFb share less than 50% sequence identity, they both maintain the ability to activate the coagulation cascade. This also demonstrates the strength of the zebrafish for studying *in vivo* biological functions of TF that are difficult to test due to mammalian lethality. With these data, we were also able to identify evidence of TF subfunctionalization. TFa is necessary and sufficient for normal venous clot formation in larvae, but only plays a minor role in the arterial system. Conversely, TFb is necessary and sufficient for an intact arterial response to injury while occupying a lesser function in the venous system. The 10 to 100-fold greater procoagulant activity of TFa over TFb in the *ex vivo* trout plasma assay likely explains that the dominant venous function is due to a functional difference between TFa and TFb, but also suggests a distinct mechanism for the enhanced arterial activity of TFb. Conversely, spontaneous thrombosis in our *at3*-null model was more dependent on TFb rather than TFa. This raises the possibility of differential behaviors for the two types of TF activity in hemostatic versus thrombotic conditions. For example, graft failure due to thrombosis is a significant clinical problem and several studies implicate ectopic TF expression in response to hemodynamic changes as a driver of clot formation. [52–54] Our model suggests differential behaviors for the two TFs might inform specific and novel methods for modifying spontaneous thrombosis in the venous and arterial circulation.

Unfortunately, spatiotemporal studies for *f3* expression have been unsuccessful presumably due to the low absolute levels of *f3* mRNAs and the lack of available antibodies. [55] Two large single-cell transcriptome datasets find that *f3a* has higher expression in the ventral aspect of the embryo near the venous circulation while *f3b* is more broadly expressed. [56, 57] Interestingly, evidence for *f3b* expression in hemangiogenic progenitors points to a potential function in cell differentiation that may partially explain the observed arterial phenotype. [56] Future work to understand the precise cell types where the *f3* ohnologs are expressed will help sort out their disparate roles.

Although FXIa-mediated activation of FIX is well known [58, 59], it is also established that FIX is an important substrate of the TF:FVIIa complex. [60, 61] In fact, a mutation in FIX that interferes with its interaction with TF has been shown to cause mild hemophilia. [62] Zebrafish do not have known orthologs for FXI or FXII, suggesting that TF could be the primary activator of zebrafish FIX. [63] We hypothesize that TFa:FVII and TFb:FVII complexes may have different affinities for FIX and FX, with distinct roles in the extrinsic or intrinsic pathways. Although zebrafish have 2 copies of FIX (*f9a* and *f9b)*, transcriptomic profiles of early development show that *f9b* is the predominant form prior to 5 dpf. [55] Therefore, we chose to focus on *f9b* for this study. Multigenic *f3*/*f9b* knockouts demonstrate similar patterns in both induced venous and arterial thrombus formation, more pronounced in the latter. The results suggest that TFb serves as the primary activator of intrinsic tenase, while TFa fills the canonical role of FX activation. The mild increase in TFa-mediated clot formation in the absence of FIX implies that FIX interacts with TFa and lowers the overall extrinsic tenase activity. Recently, it has been suggested that *f9a* plays a significant role *ex vivo* and at later ages. This may explain the incomplete phenotypic penetrance in this study. [64] Regardless, our work points to a role for the intrinsic pathway in mediation and a target for management of acute spontaneous thrombosis.

The ability of zebrafish to survive complete TF deficiency into early adulthood is compatible with previous studies of common pathway knockouts, [25–28], with all showing spontaneous intracranial and pericardial hemorrhage, a pattern similar to the adult mouse deletion of prothrombin. [65] The resistance to developmental lethality may be due to the low stress aquatic environment, the lack of birthing trauma, low-pressure vasculature, or species-specific differences in hemostatic factors. By chemically inducing stress, we were able to elicit a phenotype in TF-deficient larvae consistent with cardiac tamponade, which might be indicative of an inducible vascular defect. This same phenotype was observed in the *f2* knockout, but not with loss of fibrinogen. The implication is the presence of secondary functions for TF and thrombin that are distinct from fibrin polymerization, e.g. thrombocyte regulation, PAR signaling, and/or integrin binding [6–8]. These data suggest a possible inducible vascular defect that leads to hemorrhage. Notably, cardiac-specific *f3* knockout mice demonstrate a mild hemostatic defect but significant fibrosis and hemosiderosis when chemically challenged with isoproterenol, and humanized low-TF mice similarly show significant cardiac fibrosis and left ventricular dysfunction in adulthood. [9, 17] Alternative explanations include subtle cardiac-specific structural abnormalities and/or changes in vascular development such as vascular remodeling that were beyond the detection of our vascular survey.

Overall, the duplication of *f3* in zebrafish and the evidence for subfunctionalization offers multiple opportunities to explore the subtleties of TF function. The work presented is consistent with the expectations from previous genetic knockouts of procoagulant factors in zebrafish. However, the novel differentiation of arterial and venous functions and potential substrate specialization facilitates asking narrower questions than previously addressable in mammals.

## Materials and methods

### Ethics statement

Zebrafish maintenance and use was according to protocols approved by the University of Michigan Institutional Animal Care and Use Committee, identification number PRO00010679.

### Animal care

Wild-type fish were a hybrid line generated by crossing AB and TL zebrafish acquired from the Zebrafish International Resource Center. Genetic knockouts of prothrombin (*f2)* and fibrinogen alpha (*fga)* were generated and described previously [26, 28]. Tricaine (Western Chemical) was used for anesthesia and rapid chilling in an ice water bath for euthanasia.

### Targeted mutagenesis using genome editing

Throughout the text, we use “TF” and “FIX” to refer to the tissue factor and factor IX proteins, and “*f3*” and “*f9b*" to refer to the genes, respectively. The ChopChop2 server was used to identify predicted high efficiency target sites for CRISPR/Cas9 engineering. [66] Three guides each were selected for *f3a*, *f3b* and *f9b*. DNA oligonucleotides for the target sequence were synthesized with homology to a second common backbone oligo (S1 Table). The pieces were assembled via PCR and purified using phenol:chloroform. Single guide RNAs (sgRNAs) were synthesized using the Maxiscript T7 kit and purified via ethanol/ammonium acetate precipitation. 600 ng of each sgRNA was mixed with 0.5 uL EnGen Cas9 protein (New England BioLabs) at a 1.5:1 molar ratio in 300 mM KCl. The solution was incubated at 37°C for 10 minutes and 2 nL injected into the yolk of single cell embryos. Test embryos were lysed at 24 hpf and flanking primers used to amplify the target site. High resolution gel electrophoresis was performed to validate efficient cleavage. Larvae injected with 2 known successful guides were raised, bred, and their offspring screened for large deletions. Lines were established from single heterozygous founders. The *f9b* line is a large deletion from exon 2 to 8 (total 8 exons), so there is essentially no remaining coding sequence (Lavik and Shavit et al, in preparation).

### Genotyping of mutant offspring

Whole embryos or adult fin biopsies were lysed in buffer (10 mM Tris-HCl, pH 8.0, 2 mM EDTA, 2% Triton X-100, 100 μg/mL proteinase K) for 2 hours at 55°C followed by incubation at 95°C for 5 minutes. One microliter was used as template for gene specific polymerase chain reactions (S1 Table) and analyzed by gel electrophoresis.

### Real time quantitative polymerase chain reaction (RT-qPCR)

RT-qPCR was performed as described previously. [26] Briefly, total RNA was extracted from 4 days post fertilization (dpf) larvae using the RNeasy Mini Plus kit (Qiagen) and transcribed with oligo (dT)_12-18_ primer and Superscript III (Invitrogen). Three equal pools of whole larvae were tested per genotype. The resulting cDNA was template for qPCR (StepOnePlus, Applied Biosystems) with gene specific PrimeTime probe-based qPCR Assays (Integrated DNA Technologies) (S1 Table). The expression levels of *f3a and f3b* were normalized to the *actb2* gene and significance analyzed using the double delta Ct method as described. [67]

### Laser-induced vascular endothelial injury

Vascular endothelial injury was performed as previously described. [24, 29] Briefly, larvae were mounted in 0.8% low melt agarose and visualized using an Olympus IX73 microscope with attached Micropoint focusing system and pulsed-dye laser (Andor). 99 pulses were administered to the ventral surface of the posterior cardinal vein 5 somites caudal to the anal pore at 2 or 3 dpf or 3–5 somites caudal to the anal pore on the dorsal surface of the dorsal aorta at 2, 3, or 5 dpf. At 2 minutes, larvae were examined for formation of an occlusive thrombus and scored as either occluded or non-occluded by a blinded observer prior to genotyping. Venous injury was performed at 3 dpf, while arterial studies were done at 5 dpf since that is when thrombocytes first appear in circulation in substantial numbers. [43]

### Spontaneous thrombosis assay

Tg(*fabp10a*:*fgb-eGFP)* larvae were raised to 5 dpf and screened for transgene status by presence of signal in the liver. Larvae were scored via direct visualization of the venous circulation under high-powered fluorescent microscopy. The degree of thrombosis was scored by a single blinded observer on a scale of 0–3 with 0 being no signal and 3 being diffuse, intense signal, similar to previous work. [29] During analysis, scores ≤1 were considered negative and scores ≥2 were labeled positive.

### Membrane bound recombinant TF production

The cDNA sequences for zebrafish *f3a* and *f3b* were identified from genomic reference sequence, [68] then cloned and confirmed by Sanger sequencing. Nucleotides coding for the predicted leader peptide and cytoplasmic tail were removed. A *pelB* leader peptide (to direct expression to the *E*. *coli* inner membrane) and an HPC4 epitope tag were added (S2 Table). The resulting nucleic acid sequence was codon optimized for *E*. *coli* expression and synthesized as a gBlock (IDT, Coralville, Iowa), subcloned into the pET-26b (+) expression vector, and the validated plasmids transformed into BL21 (DE3) competent cells. For expression, transformed *E*. *coli* were grown overnight at 37°C in minimal MDG medium [69] plus 100 μg/mL kanamycin. The next day, 50 μL culture were diluted to 1 mL with water and the A_600_ measured (result multiplied by 20 to calculate A_600_ of undiluted culture). *E*. *coli* cells were collected by centrifugation (15 minutes at 6,000*g*), and then resuspended in BYE medium (50 g/L Bacto yeast extract, 12.75 mM KH_2_PO_4_, 54 mM K_2_PO_4_, 0.5% w/v glycerol, 100 μg/mL kanamycin) to achieve an estimated A_600_ of 4.5, and shaken at 25°C for 2 hours. IPTG was then added to 100 μM and cultures allowed to shake for another 4 hours, after which cells were collected by centrifugation and stored frozen at −80°C.

Frozen pellets were resuspended in lysis buffer (30 mM HEPES pH 7.4, 50 mM NaCl, 0.1% NaN_3_, 1% Triton X-100 and 0.1% lysozyme) and briefly sonicated on ice. The bottle was rocked for 15 minutes at room temperature and then CaCl_2_ added to a final concentration of 2 mM and incubated on ice for 30 minutes. The remaining debris were removed by centrifugation, and the supernatant transferred to a new container. Equilibrated Q-Sepharose beads were added to the supernatant (1g/10mL) and rocked for 1 hour at 4°C. Supernatant was passed through an equilibrated HPC4 affinity column [70]. The column was washed (30 mM HEPES pH 7.4, 1% Triton X-100, 1 M NaCl, 1 mM CaCl_2_, and 0.1% NaN_3_) and eluted (30 mM HEPES pH 7.4, 1% Triton X-100, 100 mM NaCl, 5 mM EDTA, and 0.1% NaN_3_). The resulting eluate was dialyzed against HBS-Triton X-100 (30 mM HEPES pH 7.4, 1% Triton X-100, 100 mM NaCl, and 0.1% NaN_3_) 3 times over 2 days.

The purified TF was incorporated into 80:20 phosphatidylcholine:phosphatidylserine (PC:PS) liposomes at a molar ratio of 15,000:1 (phospholipid:TF) using deoxycholate and Biobeads SM2 as previously described [71].

### Ex vivo clotting assays

Lake trout blood (courtesy of Kevin Keeler at the United States Geological Survey, Ann Arbor, MI) was collected by venous puncture and anticoagulated using 9:1 plasma:3.2% sodium citrate and centrifuged at 1500 x *g* for 15 minutes to isolate thrombocyte-poor plasma. Plasma, TF liposomes, and 25 mM CaCl_2_ were mixed at a 1:1:1 ratio and placed on a rocker at 25°C. The tubes were continually observed through repeated tilting until the solution became solid and a clot was apparent. Clots were scored by a blinded observer continuously for 5 minutes and then checked again at 15 minutes.

### Chemical treatment

Larvae were placed in a light protected plate and stock solutions of epinephrine and hydrocortisone in DMSO were added to a final concentration of 25 μM epinephrine, 0.01% hydrocortisone, 0.1% DMSO, and incubated in the dark at 28.5°C.

### Computational resources and statistical analysis

Protein structure, conservation and modifications were predicted using Clustal Omega, [72] NetNGlyc, [73] NetPhos 3.1 [39] and TMHMM. [74] Occlusion data and *ex vivo* clotting assays were analyzed using Mann-Whitney *U* or binomial proportion tests. Significance testing, graphs, and survival curves were produced with Prism (Graphpad Software, California).

## Supporting information

S1 Fig Alignment of human TF to TFa and TFb. (DOCX)

S2 Fig Complete data set of laser injury on offspring from *f3 Aa/Bb;f9b*^*+/-*^ incrosses. (DOCX)

S1 Table Oligonucleotides used in methods. (DOCX)

S2 Table Synthesized recombinant TF gBlocks. (DOCX)

S1 Video Control larval cardiac motion at 4 dpf. (MP4)

S2 Video Larval cardiac tamponade at 4 dpf. (MP4)
