# Supplementary material for: Genetic duplication of tissue factor reveals subfunctionalization in venous and arterial hemostasis
Source: PLoS Genet. 2022 Nov 30;18(11):e1010534. doi: 10.1371/journal.pgen.1010534 (PMC9744294; doi:10.1371/journal.pgen.1010534)
Supplement: S1 Fig — (DOCX) [file pgen.1010534.s001.docx]

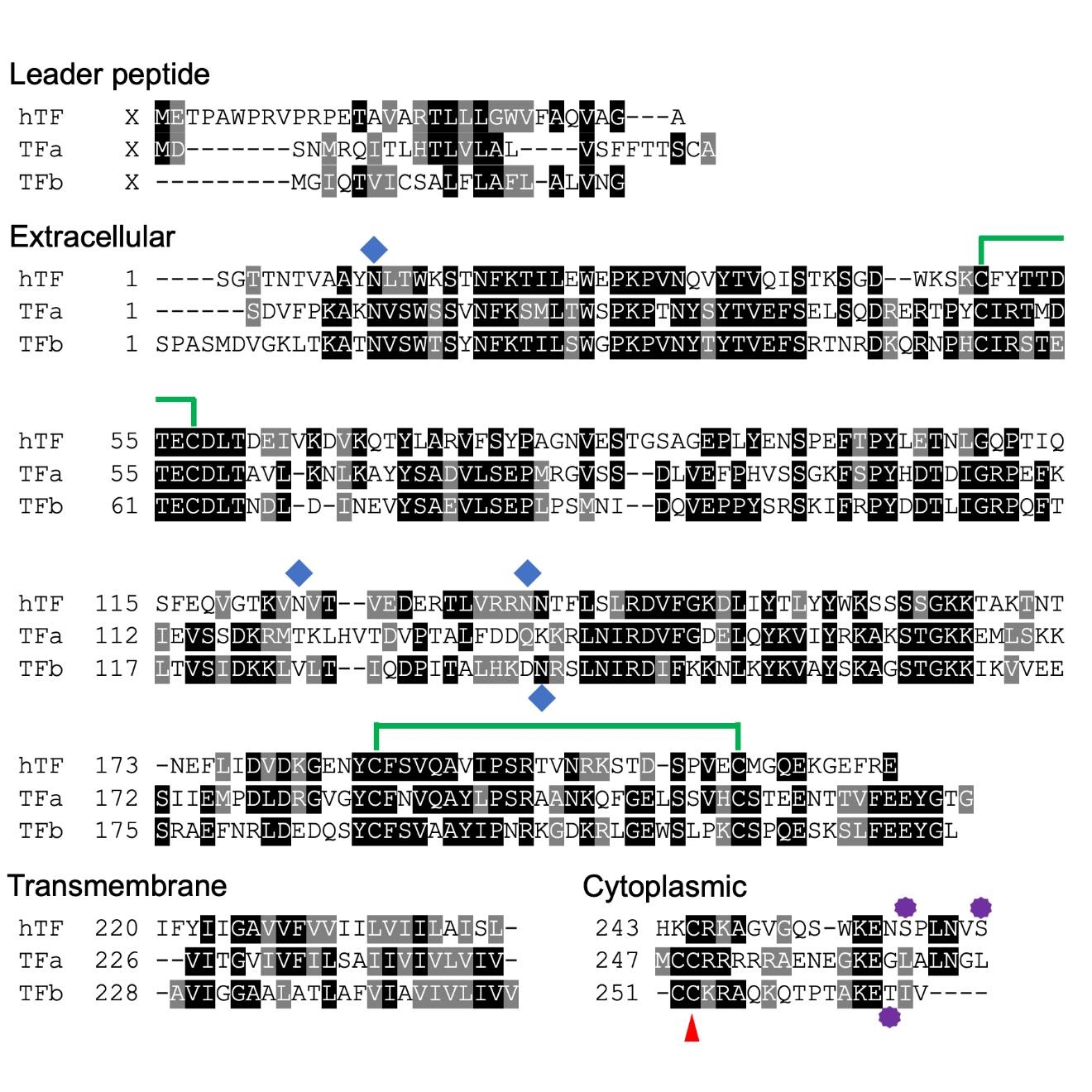


S1 Fig. Alignment of human TF to TFa and TFb.

Clustal Omega aligned sequences are shown, including annotation of the leader peptide, extracellular domain, transmembrane domain, and cytoplasmic tail. Numbering begins at the first amino acid after the propeptide. Homology is represented according to the Boxshade program with black representing identity and gray representing similarity. Sites of glycosylation (Asn11, Asn 124, Asn137) are marked with blue diamonds. TFb has 1 NetNGlyc predicted glycosylation site marked. Green lines indicate conserved residues at known disulfide bridges. Red arrow indicates a palmitoylation site. Purple marks indicate potential sites of phosphorylation in human TF and TFb (per NetPhos 3.1 Server). Transmembrane domain according to DeepTMHMM server predictions.
