# Supplementary material for: Genetic duplication of tissue factor reveals subfunctionalization in venous and arterial hemostasis
Source: PLoS Genet. 2022 Nov 30;18(11):e1010534. doi: 10.1371/journal.pgen.1010534 (PMC9744294; doi:10.1371/journal.pgen.1010534)
Supplement: S2 Fig — (DOCX) [file pgen.1010534.s002.docx]

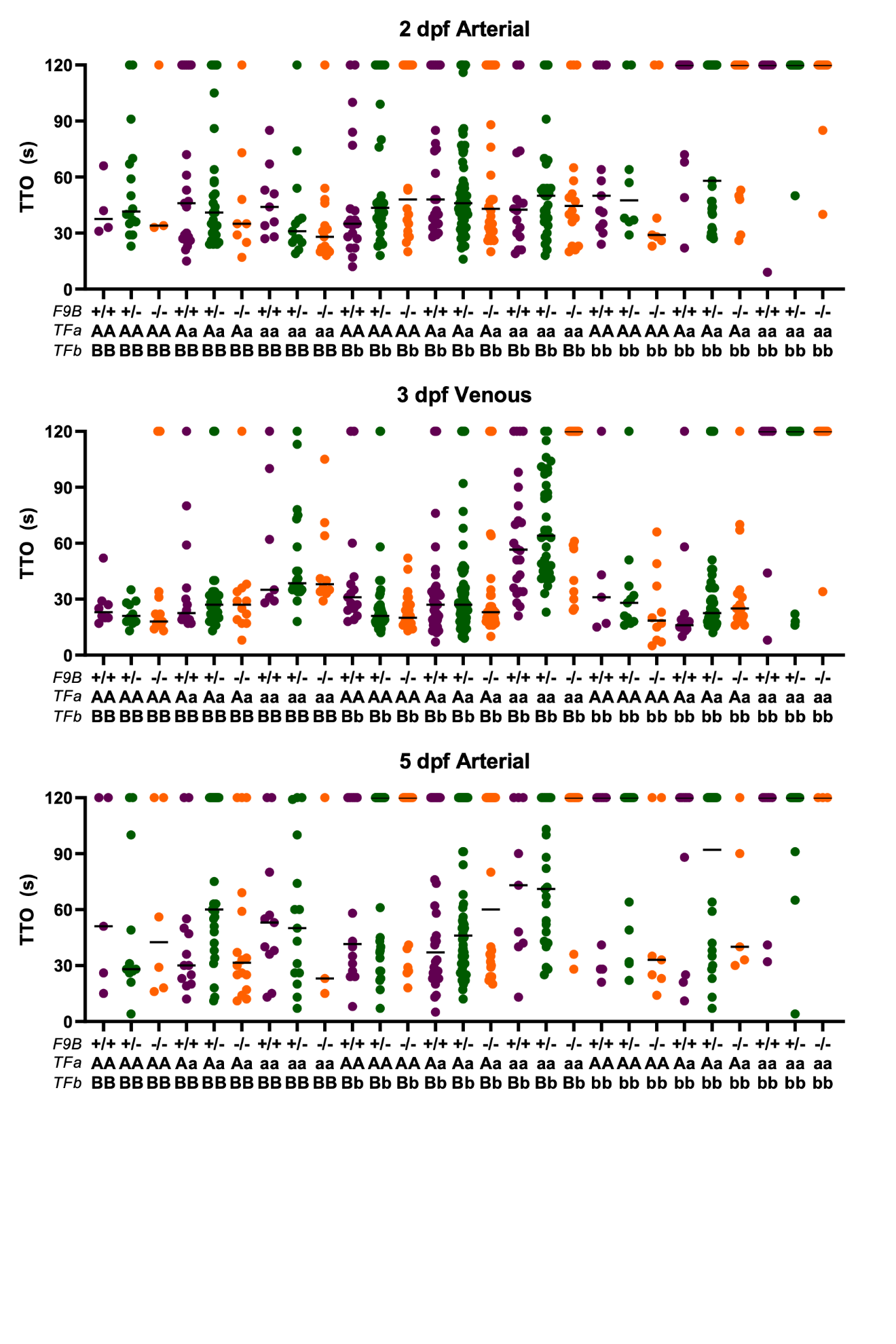


S2 Fig. Complete data set of laser injury on offspring from *f3 Aa/Bb; f9b^+/-^* incrosses.

Injury performed at 2 and 5 dpf in the arterial system and at 3 dpf in the venous system. Black lines indicate median time to occlusion. Colors serve as visual aid to distinguish *f9b^+/+^* (purple), *f9b^+/-^* (green), and *f9b^-/-^* (orange).
