# Supplementary material for: Genetic duplication of tissue factor reveals subfunctionalization in venous and arterial hemostasis
Source: PLoS Genet. 2022 Nov 30;18(11):e1010534. doi: 10.1371/journal.pgen.1010534 (PMC9744294; doi:10.1371/journal.pgen.1010534)
Supplement: S1 Table — (DOCX) [file pgen.1010534.s003.docx]

S1 Table. Oligonucleotides used in methods

| Primer Description | Primer Sequence | |
| --- | --- | --- |
| *f3a* CDS Forward | | ACACCATGGACAGTAACATGAGAC |
| *f3a* CDS Reverse | | CGCGATCGATGTCTAACATATCTACATTCTG |
| *f3b* CDS Forward | | CAACCATGGGAATTCAGACTGTA |
| *f3b* CDS Reverse | | CGCGATCGATAGGCTGTTCTGCTCACTC |
| *f2* qPCR Forward | | GAGGAAGCTCGAGAAGTGTTT |
| *f2* qPCR Reverse | | CCCTCTGTAGTCGCACATATTC |
| *f2* qPCR Probe | | FAM/TGGACAGGG/ZEN/TTGTTCCTTCACAGC/IABkFQ |
| *actb2* qPCR Forward | | ATGAAGATCCTGACCGAGAGA |
| *actb2* qPCR Reverse | | TCAAAGTCAAGGGCCACATAG |
| *actb2* qPCR Probe | | FAM/ACCACCACA/ZEN/GCTGAGAGGGAAATT/IABkFQ |
| *f3a* qPCR Forward | | GCCAAGAGCACAGGAAAGAA |
| *f3a* qPCR Reverse | | GCAGCACGTGAAGGTAGATAAG |
| *f3a* qPCR Probe | | /56-FAM/ATTGAGATG/ZEN/CCGGATTTGGACCGA/3IABkFQ/ |
| *f3b* qPCR Forward | | GACCCAAACCTGTCAACTACA |
| *f3b* qPCR Reverse | | ACTCTGTCTCTGTGCTTCTAATG |
| *f3b* qPCR Probe | | /56-FAM/TCGTGACAA/ZEN/ACAGAGAAACCCTCACTG/3IABkFQ/ |
| gRNA T7 Universal Forward | | GCTAATACGACTCACTATAGG |
| gRNA Universal Reverse | | AAAAGCACCGACTCGGTGCC |
| gRNA Backbone | | GTTTTAGAGCTAGAAATAGCAAGTTAAAATAAGGCTAGTCCGTTATCAACTTGAAAAAGTGGCACCGAGTCGGTGCTTTT |
| *f3a* gRNA #1 | | TAATACGACTCACTATAGGATACGCTTGTATTGGCGCGTTTTAGAGCTAGAAATAGC |
| *f3a* gRNA #2 | | TAATACGACTCACTATAGGCCTGTCTGAACCCATGCGGTTTTAGAGCTAGAAATAGC |
| *f3b* gRNA #1 | | TAATACGACTCACTATAGGCACAGGAAAAGTAAGTAAGTTTTAGAGCTAGAAATAGC |
| *f3b* gRNA #2 | | TAATACGACTCACTATAGGGTTACTGCTTCAGTGTAGGTTTTAGAGCTAGAAATAGC |
| *f3a* genotyping primer 1 | | AGACTCAGCCAGGACAGAGAAC |
| *f3a* genotyping primer 2 | | GAGAATTTCCCAGAGCTGACAT |
| *f3a* genotyping primer 3 | | CAGTATTGTGGACTTGGGACAA |
| *f3b* genotyping primer 1 | | TGACCATCCAAGACCCCATCA |
| *f3b* genotyping primer 2 | | CATCCCTGCGGTACATTTATTT |
| *f3b* genotyping primer 3 | | TAGGGGTTCAGCTTACCTTCAA |
| *f9b* genotyping primer 1 | | CCTCAAAGTAGCACCACTCC |
| *f9b* genotyping primer 2 | | CAATAGGTGAACATGACGTC |
| *f9b* genotyping primer 3 | | CCAGGCAGATAGGTACAGCG |
| *at3* genotyping Forward | | ACACGGAAACGAGGAATCTG |
| *at3* genotyping Reverse | | TGCAAAAATTCCTGAGGACAA |
