# Supplementary material for: Genetic duplication of tissue factor reveals subfunctionalization in venous and arterial hemostasis
Source: PLoS Genet. 2022 Nov 30;18(11):e1010534. doi: 10.1371/journal.pgen.1010534 (PMC9744294; doi:10.1371/journal.pgen.1010534)
Supplement: S2 Table — (DOCX) [file pgen.1010534.s004.docx]

S2 Table. Synthesized recombinant TF gBlocks

| >Zebrafish *f3a* CDS sequence |
| --- |
| ATGGACAGTAACATGAGACAAATTACATTACATACGCTTGTATTGGCGCTGGTTTCATTCTTCACGACGTCTTGCGCCTCAGACGTGTTTCCAAAAGCAAAGAATGTATCCTGGTCTTCTGTAAACTTTAAATCCATGCTAACGTGGAGTCCAAAACCAACCAATTATTCCTACACAGTTGAGTTTTCTGAACTCAGCCAGGACAGAGAACGCACGCCATACTGCATCAGGACGATGGATACCGAATGTGACCTGACTGCAGTGTTAAAAAACCTGAAGGCCTATTACAGCGCTGACGTCCTGTCTGAACCCATGCGGGGCGTCTCCTCTGATCTGGTCGAATTCCCTCATGTCAGCTCTGGGAAATTCTCTCCTTATCATGACACGGACATTGGTAGACCAGAGTTTAAAATAGAAGTAAGCAGCGATAAAAGAATGACAAAGCTGCATGTGACGGACGTCCCGACGGCTCTGTTTGATGATCAGAAAAAGAGATTAAATATTCGGGATGTTTTCGGGGATGAGCTGCAGTATAAGGTTATCTACAGAAAGGCCAAGAGCACAGGAAAGAAAGAAATGCTTAGTAAAAAAAGCATAATTGAGATGCCGGATTTGGACCGAGGAGTGGGTTACTGTTTTAATGTTCAAGCTTATCTACCTTCACGTGCTGCAAACAAACAATTCGGAGAGCTCAGCAGCGTCCACTGCTCTACAGAAGAAAACACAACCGTCTTTGAAGAGTACGGCACGGGTGTCATCACTGGAGTTATTGTTTTCATCCTTTCGGCAATAATAGTCATCGTTTTGGTCATCGTGATGTGCTGCAGACGAAGGAGGAGAGCGGAAAACGAAGGAAAAGAGGGATTAGCATTGAATGGTCTGTGA |
| >Zebrafish TFa protein sequence |
| MDSNMRQITLHTLVLALVSFFTTSCASDVFPKAKNVSWSSVNFKSMLTWSPKPTNYSYTVEFSELSQDRERTPYCIRTMDTECDLTAVLKNLKAYYSADVLSEPMRGVSSDLVEFPHVSSGKFSPYHDTDIGRPEFKIEVSSDKRMTKLHVTDVPTALFDDQKKRLNIRDVFGDELQYKVIYRKAKSTGKKEMLSKKSIIEMPDLDRGVGYCFNVQAYLPSRAANKQFGELSSVHCSTEENTTVFEEYGTGVITGVIVFILSAIIVIVLVIVMCCRRRRRAENEGKEGLALNGL |
| >Zebrafish *f3b* CDS sequence |
| ATGGGAATTCAGACTGTAATATGTTCGGCACTTTTCCTTGCTTTTCTTGCTCTTGTAAACGGATCTCCAGCATCAATGGATGTGGGCAAACTTACCAAAGCAACAAATGTTTCATGGACATCTTACAATTTCAAAACAATCCTGTCTTGGGGACCCAAACCTGTCAACTACACATATACAGTTGAATTCTCAAGAACAAATCGTGACAAACAGAGAAACCCTCACTGCATTAGAAGCACAGAGACAGAGTGCGACTTGACCAATGATTTGGACATAAATGAGGTTTATTCGGCCGAGGTGCTCTCAGAGCCCCTGCCCAGCATGAATATCGACCAAGTGGAGCCTCCATATAGCAGATCAAAGATCTTCCGCCCTTATGATGACACTTTAATAGGAAGACCTCAGTTCACACTGACGGTGAGCATAGACAAGAAGCTTGTGCTGACCATCCAAGACCCCATCACGGCTCTGCACAAAGACAACAGATCTTTGAACATCCGTGACATCTTTAAGAAAAATCTTAAGTACAAAGTTGCCTACAGCAAAGCTGGAAGCACAGGAAAAAAAATCAAAGTAGTCGAGGAAAGCCGAGCGGAGTTTAACCGACTGGATGAGGATCAGAGTTACTGCTTCAGTGTAGCGGCTTACATTCCTAACCGGAAAGGAGACAAGAGGCTCGGAGAGTGGAGCCTTCCCAAATGTTCACCGCAGGAAAGTAAAAGTCTGTTTGAAGAGTATGGACTGGCTGTGATTGGTGGAGCAGCGCTCGCAACCCTGGCTTTTGTGATTGCTGTAATCGTTCTGATTGTGGTGTGCTGTAAACGAGCACAAAAACAAACGCCCACAGCCAAGGAAACCATAGTCTGA |
| >Zebrafish TFb protein sequence |
| MGIQTVICSALFLAFLALVNGSPASMDVGKLTKATNVSWTSYNFKTILSWGPKPVNYTYTVEFSRTNRDKQRNPHCIRSTETECDLTNDLDINEVYSAEVLSEPLPSMNIDQVEPPYSRSKIFRPYDDTLIGRPQFTLTVSIDKKLVLTIQDPITALHKDNRSLNIRDIFKKNLKYKVAYSKAGSTGKKIKVVEESRAEFNRLDEDQSYCFSVAAYIPNRKGDKRLGEWSLPKCSPQESKSLFEEYGLAVIGGAALATLAFVIAVIVLIVVCCKRAQKQTPTAKETIV |
| >Zebrafish Membrane Localized *f3a* for E.coli (termini modifications underlined) |
| GGAGATATACATATGAAATATCTGCTGCCGACGGCGGCCGCGGGATTACTGTTGTTAGCCGCTCAGCCCGCGATGGCTGCAGAAGACCAGGTGGATCCTCGGTTGATAGATGGTAAGGTCTTTCCCAAAGCTAAGAACGTCTCTTGGAGTAGCGTTAACTTCAAATCTATGCTGACATGGTCTCCTAAGCCAACAAATTACTCGTACACGGTGGAGTTCTCGGAGCTGGGTCAGGACCGCGAGCGGACTCCCTACTGTATTCGGACAATGGATACCGAGTGTGATCTTACGGCGGTCTTAAAGAACTTAAAGGCCTATTATTCCGCTGATGTACTTAGCGAGCCGATGAGAGGTGTATCAAGCGATCTTGTTGAGTTTCCCCACGTCTCTTCTGGAAAGTTCAGTCCCTATCACGATACTGACATCGGACGTCCAGAGTTTAAGATTGAGGTCTCCTCCGATAAACGGATGACCAAACTGCATGTCACCGACGTACCTACGGCGTTATTTGATGATCAAAAAAAACGGCTTAACATCAGAGATGTTTTCGGTGACGAGTTGCAGTATAAAGTCATTTACCGTAAAGCCAAAAGCACCGGCAAGAAGGAAATGCTGTCCAAACAATCCATCATAGAGATGCCGGATTTAGACAGAGGTGTGGGATATTGTTTTAACGTGCAGGCCTATCTGCCTAGTCGCGCAGCAAACAAGCAATTTGGGGAGCTGTCCTCGGTTCATTGCTCCACCGAGGAAAATACGACGGTCTTTGAAGAATATGGGACTGGAGTGATCACCGGTGTTATCGTATTCATATTATCGGCCATAATCGTCATTGTTTTAGTAATTGTGCACAAGTGATAAGCTTTCCGACATCACCATCACCATCACTGAGATCCGGCTGCTA |
| >Translated Membrane Localized TFa (termini modifications underlined) |
| MKYLLPTAAAGLLLLAAQPAMAAEDQVDPRLIDGKVFPKAKNVSWSSVNFKSMLTWSPKPTNYSYTVEFSELGQDRERTPYCIRTMDTECDLTAVLKNLKAYYSADVLSEPMRGVSSDLVEFPHVSSGKFSPYHDTDIGRPEFKIEVSSDKRMTKLHVTDVPTALFDDQKKRLNIRDVFGDELQYKVIYRKAKSTGKKEMLSKQSIIEMPDLDRGVGYCFNVQAYLPSRAANKQFGELSSVHCSTEENTTVFEEYGTGVITGVIVFILSAIIVIVLVIVHK |
| > Zebrafish Membrane Localized *f3b* for E.coli (termini modifications underlined) |
| GGAGATATACATATGAAATATCTGCTGCCGACGGCGGCCGCGGGATTACTGTTGTTAGCCGCTCAGCCCGCGATGGCTGCAGAAGACCAGGTGGATCCTCGCCTTATTGATGGGAAAGCTAGCATGGACGTTGGCAAGTTAACTAAAGCAACGAATGTAAGCTGGACATCCTACAATTTCAAGACGATCTTGAGCTGGGGCCCGAAGCCTGTCAACTATACTTACACTGTCGAGTTTTCACGGACAAACCGCGATAAGCAGCGCAACCCTCATTGTATCAGAAGCACCGAAACTGAATGCGATCTGACGAACGACCTGGATATCAATGAGGTCTATTCCGCTGAAGTGTTATCTGAGCCTTTACCGAGCATGAATATTGATCAAGTCGAACCTCCCTATAGTCGTTCAAAGATATTCAGACCATATGACGATACTTTGATCGGACGGCCACAATTTACGCTTACAGTTAGCGTGGACAAAAAATTAGTATTGACTATACAAGATCCAATAACTGCGCTTCACAAGGATAATAGATCGCTGAACATCAGAGACATCTTTAAGAAGAATTTGAAATATAAAGTGGCTTATTCTAAGGCGGGGAGCACAGGGAAGAAGATCAAAGTTGTAGAAGAAAGCCGCGCCGAGTTCAATAGATTAGATGAGGATCAATCCTACTGTTTTTCAGTAGCGGCTTATATCCCGAATCGCAAAGGGGATAAACGGTTGGGGGAATGGAGCCTGCCAAAGTGTTCGCCACAAGAATCGAAATCACTGTTTGAGGAGTATGGCTTAGCCGTAATAGGCGGCGCAGCATTAGCGACCCTTGCATTTGTAATAGCCGTTATAGTCCTTATAGTGGTCCATAAATGATAAGCTTTCCGACATCACCATCACCATCACTGAGATCCGGCTGCTA |
| >Translated Membrane Localized TFa (termini modifications underlined) |
| MKYLLPTAAAGLLLLAAQPAMAAEDQVDPRLIDGKASMDVGKLTKATNVSWTSYNFKTILSWGPKPVNYTYTVEFSRTNRDKQRNPHCIRSTETECDLTNDLDINEVYSAEVLSEPLPSMNIDQVEPPYSRSKIFRPYDDTLIGRPQFTLTVSVDKKLVLTIQDPITALHKDNRSLNIRDIFKKNLKYKVAYSKAGSTGKKIKVVEESRAEFNRLDEDQSYCFSVAAYIPNRKGDKRLGEWSLPKCSPQESKSLFEEYGLAVIGGAALATLAFVIAVIVLIVVHK |
